# The Impact of Communication Competence on Job Performance Among Registered Nurses: A Cross‐Sectional Analysis

**DOI:** 10.1155/jonm/5238925

**Published:** 2026-04-02

**Authors:** Hadeel Al-Zawahreh, Nidal Eshah, Ahmad Hussein Rayan, Islam Oweidat, Khalid Al-Mugheed, Nadiah A. Baghdadi, Sally Mohammed Farghaly Abdelaliem

**Affiliations:** ^1^ Faculty of Nursing, Zarqa University, Zarqa, Jordan, jadara.edu.jo; ^2^ Clinical Nursing Department, Faculty of Nursing, Zarqa University, Zarqa, Jordan, jadara.edu.jo; ^3^ Community and Mental Health Nursing Department, Faculty of Nursing, Zarqa University, Zarqa, Jordan, jadara.edu.jo; ^4^ College of Pharmacy, Nursing and Medical Sciences, Nursing Department, Riyadh Elm University, Riyadh, Saudi Arabia, riyadh.edu.sa; ^5^ Department of Nursing Management and Education, College of Nursing, Princess Nourah bint Abdulrahman University, P.O. Box 84428, Riyadh, 11671, Saudi Arabia, pnu.edu.sa; ^6^ Department of Nursing, Faculty of Allied Health Sciences, Kuwait University, Kuwait City, Kuwait, kuniv.edu; ^7^ Department of Nursing Administration, Faculty of Nursing, Alexandria University, Alexandria, Egypt, alexu.edu.eg

**Keywords:** communication competence, job performance, Jordan, nurses

## Abstract

**Introduction:**

Effective communication in nursing practice is directly influencing job performance, patient outcomes, and teamwork. As the healthcare landscape becomes increasingly complex, understanding the specific communication competencies that contribute to professional development and interpersonal effectiveness is essential.

**Purpose:**

to investigate the relationship between communication competence and job performance among Jordanian nurses.

**Methods:**

A quantitative, cross‐sectional study examined the link between communication competence and job performance among 113 registered nurses at Al‐Bashir and Zarqa Governmental Hospitals. Data were collected during one month, using a self‐administered questionnaire with two validated English‐language scales: The Interpersonal Communication Competence Scale (ICCS, 30 items, *α* = 0.840) and the Six‐Dimensional Scale of Nursing Performance (6‐D Scale, 52 items, *α* = 0.903). Validity was confirmed through face and construct validation and reliability via Cronbach’s alpha. SPSS v26 was used for analysis, including multiple linear regression and Kruskal–Wallis H tests to assess relationships and demographic differences.

**Results:**

The study confirmed a statistically significant and positive relationship between communication competence and job performance. In particular, the dimensions of empathy (*B* = 0.318, *p* < 0.01), self‐disclosure (*B* = 0.349, *p* < 0.01), social relaxation (*B* = 0.362, *p* < 0.01), and environmental control (*B* = 0.328, *p* < 0.01) had the most substantial impact. Demographic variables (gender, educational level, years of experience, type of unit, and shift pattern) were found to significantly influence both communication competence and job performance.

**Conclusions:**

Communication competence boosts nursing performance. Key skills like empathy and self‐disclosure enhance relationships and growth. Tailored training and further research are recommended.

## 1. Introduction

### 1.1. Background

Nurses are the backbone of healthcare systems worldwide, playing a pivotal role in ensuring patient safety, delivering quality care, and supporting the operational effectiveness of healthcare institutions [1, 2]. As global healthcare systems evolve under the pressures of rapid technological advancement, aging populations, and rising patient expectations, nurses are increasingly expected to manage more complex responsibilities while operating in resource‐constrained and high‐stress environments [3, 4]. These challenges are prompting urgent calls for strategies that support nursing performance and resilience.

Communication competence refers to a nurse’s ability to effectively exchange information, build relationships, and engage in therapeutic, interpersonal, and interdisciplinary interactions within clinical settings [5, 6]. It encompasses a wide range of skills, including empathy, assertiveness, interaction management, and the ability to communicate clearly and confidently under pressure. Effective communication not only promotes collaboration within healthcare teams but also improves clinical decision‐making, reduces errors, and fosters trust with patients and families—elements essential to high‐quality care [7].

Job performance in nursing reflects the extent to which nurses fulfill their professional duties and responsibilities. It includes both task performance such as technical clinical skills, patient monitoring, and emergency response and contextual performance, which involves leadership, teamwork, interpersonal communication, and professional development [8, 9].

Global research has consistently demonstrated a significant relationship between communication competence and nursing performance. The authors in [4] found that communication competence was the strongest predictor of nursing performance among critical care nurses in South Korea, explaining 39% of performance variance. Similarly, the authors in [2] observed strong correlations between communication competence, job satisfaction, and performance, suggesting that improving communication skills enhances both morale and job effectiveness.

In many healthcare systems, communication remains underdeveloped, particularly in regions facing chronic underfunding and workforce challenges. Structured communication training is often lacking in nursing education and professional development programs, which leaves many nurses inadequately prepared for the complex communicative demands of clinical practice [10]. In the Middle East—and Jordan in particular—these issues are even more pressing. Jordan’s healthcare system is burdened by financial constraints, outdated infrastructure, and an overstretched workforce tasked with meeting the needs of both a growing national population and large communities of refugees from neighboring countries [11]. Nurses, who constitute approximately half of the healthcare workforce, are central to delivering care in this challenging environment. However, they are frequently required to operate under high patient‐to‐nurse ratios, limited staffing, and intense time pressures [12, 13].

Despite these realities, most studies in the Jordanian context emphasized organizational, managerial, or motivational aspects of nursing performance and often tended to lack theoretical grounding or fail to employ validated measurement tools, with little attention paid to how communication competence contributes to nurses’ effectiveness on the job [12]. In Jordan, the hierarchical nature of clinical settings and traditional cultural norms such as indirect communication styles can restrict open and assertive communication among nursing staff, particularly in interdisciplinary settings [13]. The healthcare system’s ongoing challenges—including refugee influxes, staffing shortages, and high patient‐to‐nurse ratios—intensify the demand for effective communication while exacerbating related barriers [12, 14].

While communication competence is universally acknowledged as a cornerstone of effective nursing practice, its specific impact on nurses’ job performance in Jordan remains insufficiently underexplored. Existing literature in Jordan focuses primarily on factors such as leadership style, job satisfaction, organizational commitment, and workload stress as determinants of nursing performance [11, 15]. However, these studies have largely overlooked the mediating role of communication competence in core nursing responsibilities such as patient advocacy and interdisciplinary collaboration [11, 15].

To date, no comprehensive, quantitative study in Jordan has empirically measured the relationship between nurses’ communication competence and their job performance using validated tools. Therefore, the present study seeks to fill this void by examining how various dimensions of communication competence predict job performance among Jordanian nurses and whether demographic factors such as gender, educational level, and experience modify this relationship.

## 2. Aim of the Study

The purpose of this study is to investigate the relationship between self‐perceived communication competence and job performance among registered nurses working in healthcare facilities in Jordan.

## 3. Methodology

### 3.1. Study Design

This study utilized a descriptive correlational cross‐sectional design. The descriptive design allowed the researcher to measure workplace fun levels and innovative behavior among nurses working in Jordanian hospitals [16]. Meanwhile, the cross‐sectional design enabled the researcher to measure variables at a specific time, providing a snapshot of the variables as they occurred in the sample of interest [16].

### 3.2. Settings

The study was conducted in two governmental hospitals in Jordan: Al‐Bashir Hospital and Zarqa Governmental Hospital. Al‐Bashir Hospital, located in Amman, is Jordan’s largest and busiest public hospital, serving a high volume of patients, including complex and critical cases, and employing a large nursing workforce with diverse specialties. Al Bashir Hospitals, the largest public hospital in Amman, had a total bed capacity of 1,150 beds and employed 1,200 nurses.

On the other hand, Zarqa Governmental Hospital, located in Zarqa, is a regional healthcare facility that caters to a diverse patient demographic, including a high proportion of underserved populations, and has fewer specialized units. The hospital had a capacity of 500 beds and was equipped with all necessary medical equipment and specializations.

#### 3.2.1. Population and Sample

The target population comprised all registered nurses working in governmental hospitals across Jordan, while the accessible population included registered nurses employed at Al‐Bashir Hospital and Zarqa Governmental Hospital. A convenience sampling technique was employed due to its practical feasibility. Recruitment took place across multiple hospital departments and shifts to ensure representation from diverse specialties, experience levels, and work settings. Additionally, anonymity and voluntary participation were emphasized to reduce response bias and encourage honest responses.

The sample size was calculated using G∗Power software based on a regression model with 16 potential predictors. An effect size of 0.15 was selected, representing a small‐to‐moderate effect commonly used in nursing and behavioral research where multiple variables influence outcomes. The significance level (*α*) was set at 0.05 with a statistical power (1−β) of 0.80, resulting in a minimum required sample size of 143 participants to adequately detect meaningful relationships.

**FIGURE 1:**
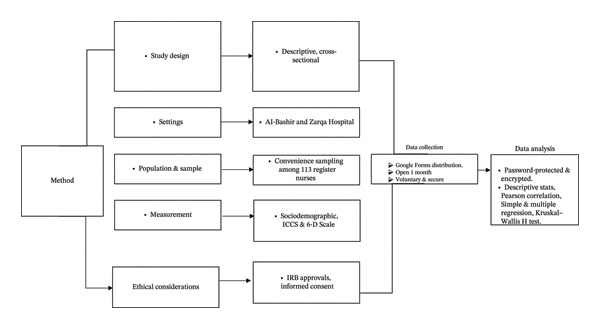
Summary of methods.

To ensure that the final sample remained adequately powered, the calculation did not originally account for potential dropouts or incomplete responses. Therefore, the recruitment target was intentionally set higher than the minimum required sample size to compensate for possible attrition and ensure that the final number of completed questionnaires met or exceeded the required sample size Figure 1.

### 3.3. Measurement

The questionnaire included 15 sociodemographic and occupational variables to examine potential factors influencing communication competence and job performance.

#### 3.3.1. Sociodemographic Variables

Age, gender, educational level, and place of residence.

#### 3.3.2. Occupational Variables

Years of nursing experience, nursing specialization, monthly income, weekly work hours, shift type, number of patients cared for per week, hospital bed capacity, participation in training programs, number of dependents, and current workload.

### 3.4. Interpersonal Communication Competence Scale (ICCS)

The ICCS tool [17] measures communication competence via 30 items across 10 subscales. Responses were recorded on a 5‐point Likert scale (1 = “Almost Never” to 5 = “Almost Always”), yielding total scores between 30 and 150, with higher scores indicating stronger competence. The ICCS has demonstrated validity through correlations with communication satisfaction (^∗^r^∗^ = 0.40), cognitive flexibility (^∗^r^∗^ = 0.49), and communication flexibility (*r* = 0.40). While the scale has not been specifically validated in Jordan, it has shown wide cross‐cultural applicability in healthcare studies conducted in countries like Turkey and South Korea [6, 18]. Cronbach’s alpha (*α*) for the combined instruments (82 items) was (*α* = 0.918 using SPSS v26. The ICCS showed high reliability (*α* = 0.840), with subscales ranging from 0.702 (empathy) to 0.801 (Supportiveness).

### 3.5. Six‐Dimensional Scale of Nursing Performance (6‐D Scale)

To assess job performance, the study utilized the 6‐D Scale developed by [19], through 52 items grouped into six dimensions. Each item is rated on a 5‐point scale (1 = Poor to 5 = Excellent), with higher scores indicating stronger perceived job performance. The scale has strong reliability (Cronbach’s α = 0.92; [20]) and validity via correlations with supervisor assessments and standard performance benchmarks. The 6‐D Scale had (α = 0.903), with subscales between 0.821 (leadership) and 0.887 (planning/evaluation). Face validity was confirmed by expert academic and practicing nurses’ review. Construct validity was supported by prior validation of the scales [17, 20].

### 3.6. Ethical Considerations

Ethical approval was granted by the IRBs of the participating hospitals and the Faculty of Nursing at Zarqa University (Approval No. 54/2024‐2025). Informed consent was obtained electronically through a Google Forms–embedded consent page, where participants voluntarily agreed by selecting “I agree to participate.” Participants were informed of their right to withdraw at any time prior to submitting the survey. Anonymity was fully ensured, as no personal identifiers were collected and IP tracking was disabled. The digital consent process was securely documented within Google Forms, providing a record of informed participation.

### 3.7. Data Collection Process

All collected data were stored in a password‐protected Google Drive folder accessible only to the principal investigator. At the end of data collection, the raw data were downloaded and backed up in an encrypted offline storage system to prevent unauthorized access or data loss. A screening question at the beginning of the survey verified that participants were registered nurses employed at Al‐Bashir Hospital or Zarqa Governmental Hospital. Only eligible respondents could proceed, and any ineligible entries were removed during data cleaning. The survey remained open for one month, and data collection concluded once the required sample size had been reached or the designated period ended.

The questionnaire was distributed to nurses through a secure Google Forms link shared via official hospital communication channels, including internal WhatsApp groups used by nursing staff and departmental email lists, depending on the hospital’s communication practices. Unit head nurses and nursing supervisors assisted in disseminating the survey link to their respective teams but were not involved in collecting or reviewing responses. Their role was limited to facilitating distribution to ensure that nurses across various departments and shifts received the invitation to participate. Participation remained entirely voluntary, with no pressure or follow‐up reminders tied to supervisors.

### 3.8. Data Management and Analysis

Data were analyzed using SPSS v26. Descriptive statistics (means, standard deviations, frequencies, and percentages) were used to summarize levels of communication competence and job performance. Pearson’s correlation analysis assessed the strength and direction of the relationship between the two variables. A simple linear regression was conducted to examine whether communication competence predicted job performance, as the model included one independent and one dependent variable. Additionally, **a** multiple linear regression was performed to evaluate how demographic and work‐related factors jointly predicted job performance. The Kruskal–Wallis H test was applied to determine whether communication competence and job performance differed significantly across demographic groups. A *p* value of < 0.05 was used as the threshold for statistical significance.

## 4. Results

### 4.1. Sociodemographic Characteristics of the Study Sample

The study included 113 nurses working in two governmental hospitals, and they completed the study questionnaire, resulting in a 78% response rate. The sample is predominantly female, with 67 participants (59.3%) compared to 46 males (40.7%). 59% of participants have less than 10 years of experience. All demographic characteristics are listed in Table 1.

**TABLE 1:** Demographic characteristics of the study sample (*n* = 113).

Demographic characteristics	*N*	%
Gender		
Male	46	40.7%
Female	67	59.3%.
**Total**	**113**	**100%**
Educational Level		
Less than undergraduate	28	25%
Collegiate	68	60%
Postgraduate	17	15%
**Total**	**113**	**100%**
Years of Experience		
Less than 5 years	28	25%
5‐less than 10 years	38	34%
10‐less than 15 years	25	22%
15 years and more	22	19%
**Total**	**113**	**100%**
Type of Unit		
Medical	35	31%
Surgical	30	27%
Intensive care unit (ICU)	20	18%
Pediatric	35	31%
**Total**	**113**	**100%**
Shift Pattern		
Day shifts only	65	58%
Night shifts only	44	39%
Rotating shifts (Day and Night)	4	4%
**Total**	**113**	**100%**

### 4.2. Descriptive Statistics for Study Variables and Dimensions

The study variables comprise two main variables: the independent variable (communication competence) and the dependent variable (job performance). Both the independent and dependent variables have subdimensions.

The findings revealed that the overall mean score for nurses’ communication competence was 3.608, with a standard deviation of ± 0.397, indicating that nurses in the sample generally perceive themselves as highly competent in interpersonal communication. The highest mean scores for dimensions of communication competence were recorded for empathy (4.251), assertiveness (4.201), and interaction management (4.150), respectively.

The overall job performance score among Jordanian nurses is 3.587, with a standard deviation of ± 0.337. This indicates that the nurses report a consistently high level of job performance across the study sample. The highest mean for dimensions of job performance was recorded for critical care (3.778), teaching/collaboration (3.776), and leadership (3.726), respectively (Table 2).

**TABLE 2:** Descriptive statistics for study variables and dimensions (*n* = 113).

Variables	Descriptive statistics		Rank
	Mean	Std. deviation	
Independent Variable: Communication Competence	3.608	±0.397	‐‐‐‐
Self‐Disclosure	4.000	±0.641	6
Empathy	4.251	±0.501	1
Social Relaxation	3.991	±0.709	7
Assertiveness	4.201	±0.513	2
Altercentrism	4.127	±0.569	4
Interaction Management	4.150	±0.488	3
Expressiveness	4.053	±0.513	5
Supportiveness	3.723	±0.783	10
Immediacy	3.847	±0.659	8
Environmental Control	3.746	±0.710	9

Dependent Variables: Job Performance	3.587	±0.337	‐‐‐‐‐
Leadership	3.726	±0.308	3
Critical Care	3.778	±0.297	1
Teaching/Collaboration	3.776	±0.274	2
Planning/Evaluation	3.302	±0.654	6
Interpersonal Relations/Communication	3.509	±0.664	4
Professional Development	3.435	±0.682	5

*Note:* A simple linear regression showed that communication competence significantly predicted job performance, F(1, 111) = 14.56, *p* < 0.001. The model explained 11.6% of the variance in job performance (*R* ^2^ = 0.116), with a moderate positive correlation (*r* = 0.341). The regression coefficient indicated a significant positive effect of communication competence on job performance (*β* = 0.341, *p* < 0.01) (Table 3).

**TABLE 3:** Linear regression of communication competence predicting job performance.

Category	Variable	B	Std. error	Beta	t	Sig.	R	*R* ^2^	F	Sig. (ANOVA)
Model Info	Dependent	Job Performance	—	—	—	—	—	—	—	—
	Independent	Communication Competence	—	—	—	—	—	—	—	—

Coefficients	Constant	2.543	0.275	—	9.235	0.000	—	—	—	—
	Communication Competence	0.289	0.076	0.341	3.816	0.000	0.341	0.116	14.561	0.000

*Note:* Multiple regression analyses indicated that communication competence dimensions significantly predicted all job performance domains. The strongest effect was observed for leadership (*R* = 0.827, *R* ^2^ = 0.684, *p* ≤ 0.01), followed by critical care (*R* = 0.776, *R* ^2^ = 0.602, *p* < 0.001), and teaching/collaboration (*R* ^2^ = 0.440, *p* < 0.001). Communication competence also significantly predicted planning/evaluation (*R* ^2^ = 0.208, *p* < 0.001), interpersonal relations/communication (*R* ^2^ = 0.177, *p* < 0.001), and professional development (*R* ^2^ = 0.097, *p* = 0.001). Key predictors varied by domain, including environmental control, interaction management, self‐disclosure, empathy, and social relaxation (Table 4).

**TABLE 4:** Significant communication competence dimensions’ predictors for job performance subdimensions.

Predictor	Professional development	Relations/communication	Planning/evaluation	Teaching/collaboration	Leadership	Critical care
Self‐Disclosure	0.001	0.009	0.001	—	—	—
Empathy	0.001	0.001	0.001	0.002	0.007	—
Social Relaxation	0.001	0.001	—	—	0.001	—
Assertiveness	—	—	—	0.001	—	—
Altercentrism	—	—	0.004	0.007	—	0.023
Interaction Management	0.040	0.050	0.021	0.000	0.001	0.008
Expressiveness	—	—	—	0.036	—	—
Supportiveness	—	0.042	—	0.004	—	0.028
Immediacy	—	—	0.012	—	0.041	0.030
Environmental Control	0.035	0.001	0.033	—	0.001	0.001

Significant differences in communication competence and job performance were observed among Jordanian nurses based on demographic characteristics. Female nurses had higher mean ranks than males (communication competence: 51.79 vs. 42.20; job performance: 51.99 vs. 42.01, *p* < 0.001). Postgraduate nurses scored highest compared to bachelor’s and diploma holders (communication competence: 79.00; job performance: 81.15, *p* = 0.001/< 0.001). Nurses with ≥ 15 years of experience had the highest scores (communication competence: 80.36; job performance: 77.30, *p* < 0.001). Medical unit nurses scored highest (communication competence: 79.15; job performance: 76.09), whereas ICU nurses scored lowest (28.25; 26.31, *p* < 0.001). Day‐shift nurses showed the highest mean ranks (communication competence: 41.01; job performance: 42.21), and rotating‐shift nurses the lowest (12.00; 11.12, *p* < 0.001) (Table 5).

**TABLE 5:** Communication competence and job performance by demographic characteristics.

Demographic variable	Groups	Mean rank–communication competence	Mean rank–job performance	*p* value
Gender	Female	51.79	51.99	0.005
	Male	42.20	42.01	

Educational Level	Postgraduate	79.00	81.15	
	Bachelor	49.25	50.10	0.004
	Diploma	34.50	35.20	

Years of Experience	≥ 15 years	80.36	77.30	
	10–14 years	65.20	63.80	
	5–9 years	48.15	49.10	0.003
	< 5 years	31.25	32.00	

Type of Unit	Medical	79.15	76.09	
	Surgical	61.50	60.20	0.004
	ICU	28.25	26.31	
	Other Units	45.10	43.85	

Shift Pattern	Day Shift	41.01	42.21	
	Rotating Shift	12.00	11.12	0.002
	Night Shift	27.50	28.00	

## 5. Discussion

The analysis indicates that Jordanian nurses perceive themselves as having a high level of communication competence overall. This suggests a strong and consistent ability to apply essential communication behaviors in daily interactions with patients, colleagues, and multidisciplinary healthcare teams. Such competence is vital in clinical settings, where effective communication can significantly influence patient outcomes, team collaboration, and overall job performance. The higher levels observed in this study may reflect the growing emphasis on communication training in Jordanian nursing education and professional development programs [21].

Within the specific subdomains of communication competence, nurses demonstrated particular strength in empathy, followed by assertiveness and interaction management. This indicates that Jordanian nurses possess well‐developed interpersonal skills, especially in showing empathy—an essential attribute in nursing practice. Assertiveness and interaction management further support clear, respectful communication and effective coordination in complex healthcare environments [6]. These findings suggest that nurses in Jordan are well‐prepared to navigate both the emotional and operational demands of their roles.

The high level of job performance among nurses aligns with findings by [22], who also reported high performance levels among nurses in similar healthcare settings. Such consistency reflects a competent and reliable nursing workforce capable of effectively meeting role demands. The highest scores were in critical care tasks, highlighting nurses’ ability to respond efficiently in urgent and high‐pressure situations. In contrast, planning and evaluation, while still rated highly, emerged as comparatively less strong. This may be due to the more complex and infrequent nature of strategic planning tasks in daily practice, as opposed to the immediacy and frequency of direct clinical care. Nonetheless, this indicates a valuable opportunity to enhance performance in planning and evaluation through targeted training and professional development initiatives aimed at strengthening nurses’ strategic and reflective practice skills.

The findings confirm that communication competence significantly enhances job performance among nurses. This relationship underscores the critical role that effective communication plays in professional healthcare environments. These results align with previous research, including [23], which emphasized the direct link between communication skills and improved workplace outcomes. Similarly, [25] reinforced that effective communication fosters better performance by facilitating teamwork, reducing errors, and improving task coordination.

Specific dimensions of communication competence notably influence leadership. Among these, the ability to manage the environment, effectively navigate interpersonal interactions, and maintain social composure were key traits associated with strong leadership. The significance of empathy and immediacy also highlights the importance of emotional intelligence and responsiveness in leadership roles. These results support prior literature [23, 25], which positions communication as a foundational element of leadership success, particularly in high‐stress environments like healthcare. However, not all communication dimensions showed a significant impact, suggesting that while communication is vital, certain traits may be more influential in leadership effectiveness than others. Overall, the findings provide strong evidence that enhancing communication competence can positively influence both performance and leadership in nursing contexts.

Regarding the critical care subdimension of job performance, environmental control was the strongest predictor, suggesting that nurses who effectively manage their surroundings perform better in high‐pressure care settings. This aligns with [26], who emphasized the value of a controlled and calming environment in critical care. Interaction management and supportiveness highlight the importance of managing relationships and offering emotional support to colleagues and patients. These results are consistent with [27], who argued that supportive communication fosters better clinical teamwork and care delivery. Immediacy also played a key role, reinforcing the conclusion [28] that prompt and clear communication is essential in urgent medical situations.

Interaction management had the strongest impact on teaching and collaboration, emphasizing the value of effectively navigating interpersonal interactions in environments where teamwork and knowledge‐sharing are vital. This aligns with [29], who noted that the ability to manage interpersonal dynamics is essential for productive collaboration and teaching in clinical settings. Additionally, assertiveness and supportiveness significantly impact teaching and collaboration, suggesting that nurses who can confidently share their perspectives and provide encouragement are more likely to engage in meaningful teamwork.

Self‐disclosure, empathy, altercentrism, immediacy, and environmental control were found to significantly contribute to planning care and evaluating outcomes effectively. These findings are consistent with previous research [30], supporting the idea that these communication traits enhance collaborative decision‐making and patient‐centered evaluation.

Empathy, social relaxation, self‐disclosure, supportiveness, and environmental control all showed significant positive effects on interpersonal relations. These findings suggest that emotionally attuned, open, calm, and supportive communication—alongside the ability to manage the care environment—strengthens nurses’ interpersonal interactions with patients and colleagues. These results align with prior research [28, 30–33], which highlights the critical role these dimensions play in fostering trust and collaboration in healthcare teams.

Self‐disclosure, empathy, social relaxation, and environmental control were all found to have a significant positive influence on professional development. These dimensions enhance interpersonal communication, emotional understanding, environmental management, and openness—each of which facilitates clearer communication, stronger team integration, and more effective responses to the demands of healthcare settings. These findings align with previous research [30–33], affirming the importance of these competencies in supporting personal and career growth in nursing.

Regarding demographic variables and their impact on communication competence and job performance, the findings were consistent with several studies and reinforced the broader consensus in nursing literature, where all demographic variables significantly influence both communication competence and job performance. The authors in [34] and [22] emphasized that older nurses and those with greater clinical experience tend to exhibit higher job performance, which aligns with the study’s findings. The impact of educational attainment on performance was also supported by [25] and [24], who reported that higher education levels contribute to better leadership and planning capabilities—results echoed in the current analysis. Moreover, the influence of shift patterns, particularly the challenges associated with night and rotating shifts, was well‐documented by [22], who found that irregular working hours negatively affect nurses’ effectiveness. This corresponds with the current study, which revealed lower mean ranks in communication and performance among those working night or rotating shifts.

### 5.1. Implications and Recommendations

The study’s findings of a strong positive relationship between communication competence and job performance demonstrate that enhancing communication skills can directly improve nursing outcomes, including collaboration, clinical reasoning, leadership, and professional development. This highlights the urgent need for hospital administrators and nursing leaders to prioritize communication training as a core component of ongoing professional development programs.

Considering that nurses with higher educational levels and more years of experience demonstrate better communication skills and performance, the study suggests that nursing curricula should be reviewed to include structured, evidence‐based communication modules. These modules should go beyond theoretical instruction to incorporate simulation, role‐playing, and reflective practice. Additionally, healthcare institutions should implement systemic changes to support a communication culture within hospitals, including reducing nurse‐patient ratios, limiting excessive workload, and ensuring supportive leadership that encourages open dialogue and interdisciplinary collaboration. Exploring the longitudinal impact of communication training programs on job performance and patient outcomes is recommended for healthcare systems through further research.

### 5.2. Strengths and Limitations

The study employed validated and reliable instruments, ensuring robust measurement of communication competence and job performance. A combination of descriptive and inferential analyses allowed for a nuanced understanding of the relationships between these variables and nurses’ sociodemographic and occupational characteristics. However, several limitations should be noted. The cross‐sectional design prevents causal inferences, and reliance on self‐reported data may introduce social desirability bias. Additionally, the exclusion of private‐sector nurses limits the generalizability of the findings to all healthcare sectors in Jordan. Finally, the actual sample size (*n* = 113) was below the calculated requirement (*n* = 143), potentially reducing statistical power and the ability to detect smaller effects.

## 6. Conclusion

The study provides valuable insights into the significant relationship between communication competence and job performance among nurses in Jordan. The findings demonstrate that specific dimensions of communication competence such as empathy, interaction management, and supportiveness play a critical role in enhancing key aspects of job performance, including collaboration, interpersonal communication, and professional development. Sociodemographic and occupational factors such as gender, education level, years of experience, and shift patterns were also found to influence both communication competence and performance outcomes. These results highlight the importance of integrating structured communication training into nursing education and continuous professional development programs to strengthen the nursing workforce and improve healthcare delivery.

## Author Contributions

Hadeel Al‐Zawahreh and Nidal Eshah: conceptualization and methodology.

Ahmad Hussein Rayan and Islam Oweidat: validation and formal analysis.

Nadiah A. Baghdadi and Khalid Al‐Mugheed: writing and data curation.

Sally Mohammed Farghaly Abdelaliem: formal analysis and writing.

## Funding

This study was supported by the Princess Nourah bint Abdulrahman University Researchers Supporting Project number (PNURSP2026R293); Princess Nourah bint Abdulrahman University, Riyadh, Saudi Arabia.

## Consent

The authors have nothing to report.

## Conflicts of Interest

The authors declare no conflicts of interest.

## Data Availability

All data that were analyzed during this study are included in this article, and further inquiries can be directed to the corresponding author.
